# Heat Shock Proteins and Regulatory T Cells

**DOI:** 10.1155/2013/813256

**Published:** 2013-03-14

**Authors:** E. W. Brenu, D. R. Staines, L. Tajouri, T. Huth, K. J. Ashton, S. M. Marshall-Gradisnik

**Affiliations:** ^1^School of Medical Science, Griffith Health Institute, Griffith University, Gold Coast, QLD, Australia; ^2^National Centre for Neuroimmunology and Emerging Diseases, Griffith University, Gold Coast, QLD, Australia; ^3^Queensland Health, Gold Coast Public Health Unit, Gold Coast, QLD, Australia; ^4^Faculty of Health Science and Medicine, Bond University, Gold Coast, QLD, Australia

## Abstract

Heat shock proteins (HSPs) are important molecules required for ideal protein function. Extensive research on the functional properties of HSPs indicates that HSPs may be implicated in a wide range of physiological functions including immune function. In the immune system, HSPs are involved in cell proliferation, differentiation, cytokine release, and apoptosis. Therefore, the ability of the immune system, in particular immune cells, to function optimally and in unison with other physiological systems is in part dependent on signaling transduction processes, including bidirectional communication with HSPs. Regulatory T cells (Tregs) are important T cells with suppressive functions and impairments in their function have been associated with a number of autoimmune disorders. The purpose of this paper is to examine the relationship between HSPs and Tregs. The interrelationship between cells and proteins may be important in cellular functions necessary for cell survival and expansion during diseased state.

## 1. Introduction 

Optimal cellular function is regulated by several molecules including heat shock proteins (HSPs). These proteins have chaperone properties and are important in both stressed and unstressed cells. HSPs can be categorized into six diverse highly or less-conserved families. These include HSP10, HSP40, HSP60, HSP70, HSP90, and HSP100 [1–4]. HSP60 is found in the mitochondria [5]. HSP70 is implicated in protein transport assembly and synthesis. It has anti-apoptotic properties that are implicated in intrinsic and extrinsic apoptotic pathways. HSP70 interacts with the mitochondria through death receptor signaling where it binds to death receptors DR4 and DR5 impeding TNF-related apoptosis inducing ligand (TRAIL) [6]. Importantly, HSP70 can bind to either the unphosphorylated C terminus of protein kinase C or Akt prompting rephosphorylation and kinase stabilization [7]. ATP-dependent HSP90 regulates cell survival by stabilizing kinases such as Akt and suppressing apoptosis by inhibiting caspases [8, 9]. The exact structural domains for all human HSPs remains to be determined; however, HSP70 and HSP90 have been well characterized. HSP70 is comprised of an N-terminal nucleotide-binding domain with ATPase activity and a C terminal containing a substrate-binding domain [10–12]. HSP90 on the other hand has three characterized structural domains including an N-terminal nucleotide-binding domain, a middle segment and a C terminus [13–16]. Inter-domain interactions occur by a conserved linker [17].

HSPs are found in intracellular and extracellular spaces as well as in the circulation. Intracellular HSPs including HSP27, HSP70, and HSP90 have direct roles in preventing protein aggregation, induction of cell death pathways, cellular rescue and maintaining receptor interactions [18]. These HSPs may be overexpressed in most cancers promoting the growth and survival of tumor cells. The downregulation of their expression results in a substantial decrease in tumor cells [3, 19–21]. Extracellular HSPs, HSP70, HSP90, and gp96 are peptide carriers, inducers of cytokines, and stimulants for immune cells during stress [18]. These HSPs may be either membrane bound to the plasma membrane or released into the circulation [9]. In general, there are two sources of extracellular HSPs, pathogen- and human-derived HSPs. Pathogen derived HSPs are found in the extracellular space as a consequence of infection while human-derived HSPs are released into the extracellular space in the event of intracellular traumas such as apoptosis or necrosis [8]. Extracellular HSPs may be attached to the plasma membrane. Additionally, extracellular HSPs induce the maturation of dendritic cells and present peptide molecules to antigen-presenting cells (APCs) thus, linking the innate immune and adaptive immune systems. Cell-free or circulating HSPs, for instance, HSP70, are released into the circulation by glial cells, B cells, PBMCs, or following necrosis [22–25]. These HSPs can be found in the serum and plasma. Cytokines, interferon (IFN)-*γ* and IL-10, can cause the release of HSP70 from exosomes [26]. Thus, HSP70 may serve as molecular markers of diseases such as acute myocardial infarction where these HSPs are unduly expressed in the circulation [27]. 

In unstressed cells, HSPs are chaperone proteins that maintain protein configuration and transport. The presence of HSPs is advantageous especially during cell stress owing to the versatility in their functional attributes, encompassing the inhibition of protein processing, regulation, and production [28, 29]. In the incident of stress, the heat shock functional domain and heat shock transcription factors (HSFs), including HSF-1, HSF-2, HSF-3, and HSF-4, are activated ensuing in the stimulation of the heat shock response (HSR) [30]. HSF-1 translocates into the nucleus oligomerizes, phosphorylates, and binds to heat shock elements causing the release of RNA polymerase [31, 32]. The heat shock functional domain is comprised of a nucleotide-binding domain and a peptide-binding domain. Hydrolysis of ATP to ADP occurs when it binds to the adenine nucleotide-binding domain causing a structural change and detachment of substrates. The peptide domain interacts with the hydrophobic substrates [33]. 

A set of HSPs known as small HSPs have also been identified to have an involvement in the immune system; nonetheless, this paper is limited to HSPs and not small HSPs.

## 2. Heat Shock Proteins and the Immune System 

The immune system is an intricate network of cells and proteins, and bidirectional communication between different components of the immune system is necessary for optimal homeostasis. HSPs are implicated in both the adaptive and innate immune systems. In the innate immune system, HSPs stimulate dendritic cells and macrophages, as these are APCs, they consecutively stimulate adaptive immune cells [34, 35]. HSPs are important in NK cell function as they are known to increase cytotoxic function and cell proliferation [36]. In particular, membrane-bound HSP70 on various cancer cells is recognized by cluster of differentiation (CD)94 on the NK cell, initiating effective cytolysis of the tumor cells [37, 38]. HSPs may induce the secretion of either anti- or proinflammatory cytokines thereby monitoring the immune response [39, 40].

HSPs may serve as immunogens released in response to an inflammatory episodes which associates with particular surface receptors to induce adaptive immune reactions [39, 41–44]. Antigenic binding of HSPs occurs via interactions between hydrophobic residues such as the V436 in DnaK and bound peptides hence, mutations in these residues may obscure adaptive immunity as a consequence of loss in binding abilities of the peptide [45]. Similarly, HSPs increase the effectiveness of cross-presentation between antigens and APCs in the extracellular milieu, perpetrating in the presentation of peptides to major histocompatibility complex class one (MHCI) or MHCII molecules on T cells. CD91, an HSP receptor, is a requisite for this process and increases T-cell-mediated responses related to T helper (Th)1, Th2, and Tregs [9]. Thus, in the presence of tumors, concomitant relations between the extracellular HSPs and the APCs following internalization of the tumor peptides via CD91 pathway generate both anti-and proinflammatory immune response mediated by T cells [46].

Incidentally, extracellular HSPs may have potent cytokine-related properties necessary for immune response. They act via the association with pattern recognition receptors (PRR) including toll-like receptors (TLRs) and CD14. CD14 is a lipopolysaccharide membrane protein receptor lacking a transmembrane or an intracellular domain [47]. CD14 is a necessary stimulant for HSP60 and HSP70. Following stimulation, these extracellular HSPs are endocytosed causing calcium influx and phosphorylation [48]. Myeloid differentiation primary response gene (MYD) 88 associates with the cytoplasmic domain of the TLR while interleukin (IL)-1 receptor-associated kinase (IRAK) is recruited, phosphorylated, and released. IRAK interacts with the tumor necrosis factor (TNF) receptor-associated factor 6 (TRAF6). This is sequentially followed by the stimulation of transforming growth factor-*β*-activated protein kinase 1 (TAK1) [49]. TAK1 stimulates I*κ*B Kinase (IKK) and this phosphorylates I*κ*B prompting the movement of nuclear factor kappa-light-chain enhancer of activated B cells (NF*κ*B) to the nucleus where it binds to target genes. NF*κ*B modulates the transcription of cytokine genes including TNF-*α*, IL-1*β*, IL-6, and IL-12 [50]. The generation of NF*κ*B elicits sequences of events altering the expression of cytokines, chemokines, cell adhesion molecules, growth factors, anti-apoptotic proteins, and immune receptors [51]. HSP may repress NF*κ*B successively decreasing TNF-*α* [52]. This occurs via mitogen-activated protein kinases (MAPK) pathway originating in the phosphorylation of c-Jun, which sequentially stimulates activator protein (AP)-1 and upregulates proinflammatory IL-18. Similarly, excess secretion of IL-18 is regulated by HSR which suppresses IL-18, by inhibiting JNK/MAPK signaling [53]. 

The multifaceted nature of HSPs incorporates the regulation of reactive oxygen species (ROS) and some chemokines from stimulated monocytes, macrophages, and dendritic cells. Equally, heightened HSP antigen presentation in APCs is correlated with an increase in CD86, CD40, and MHC molecules [9, 54]. HSP60 expressively inhibits chemotaxis and in combination with TLRs upregulates anti-inflammatory reactions while altering B-cell activity [55, 56]. Involvement of HSPs in the mechanism of danger-activated molecular pattern (DAMPS) is controversial as they have been described as DAMPs while elsewhere they have been implicated in the dampening of DAMPS owing to their interactions with TLRs thus, inducing proinflammatory responses [57, 58]. DAMPS are intracellular endogenous molecules secreted following necrosis with the ability to induce nonspecific adaptive immune responses following dendritic cell activation. DAMPS may also interact with pattern recognition receptors (PRRs) resulting in the presence of inflammatory cytokines [59]. In mice, macrophages stimulated with LPS release high mobility group box 1 (HMGB1) sequestering a proinflammatory response which effectively prompts apoptosis [60]. In the presence of HSP, HMGB is not translocated to the nucleus averting induction of the apoptotic pathway; thus, cell death is aborted [61]. Similarly, HSF-1 binds to the promoter regions of cytokine genes such as TNF-*α* and IL-1*β* in mice, inhibiting TNF-*α* expression in macrophages [62]. TNF-*α* is involved in the TRAIL death receptor pathway and perhaps obscures their production preventing nonspecific cell death that may be harmful to the immune response or arouse an overreactive immune response that activates autoimmunity. 

In autoimmune diseases, HSPs may be important in regulating T-cell-related cytokine dominance from a primarily proinflammatory to an anti-inflammatory state [40, 63–65]. High incidence of HSP70 decreases endotoxin-induced protein and mRNA levels of TNF-*α* in heat-induced peritoneal macrophages [66] suggesting an influence of HSPs on the transcription of these genes. However, overexpression of HSP70 by peripheral blood macrophages decreases LPS-induced TNF-*α*, IL-1*β*, IL-10, and IL-12 [67]. Additionally, HSP70 alters proinflammatory cytokine production increasing endotoxin tolerance and survival [68].

## 3. The Role of Heat Shock Proteins in Regulatory T Cell Function 

Regulatory T cells (Tregs) are a subset of CD4^+^ T cells with suppressive functions. Two main groups of Tregs have been characterized based on their site of development, that is, in the thymus (natural Tregs (nTregs)) and in the periphery (inducible Tregs (iTregs)) [69]. NTregs are derived from bone marrow progenitor cells transported to the thymus where they differentiate into nTregs following negative and positive selection. Following maturation, these cells migrate to the periphery [70]. NTregs can be differentiated from other T cells owing to the exclusive expression of forkhead box P3 (FOXP3) which is necessary for nTreg-suppressive function [71, 72]. In mice nTregs can be differentiated from iTregs owing to the presence of high levels of neuropilin-1 on mice nTregs [73]. Other essential effector and costimulatory molecules that are expressed by these cells include CD39, CD73, cytotoxic T-lymphocyte antigen 4 (CTLA-4), lymphocyte-activation gene 3 (LAG-3), CD28, CD80/86, CD40, OX40 (CD134), and 4-IBB (CD137) [74–76]. ITregs are generated from naive CD4^+^ T cells subsequent to induction by IL-10 and TGF-*β* resulting in two populations of iTregs, type 1 Tregs (Tr1), and T helper 3 (Th3) cells, respectively [77–79]. Suppressive function of these cells occurs via IL-10 and TGF-*β*. Peripheral Tregs can also be generated through interactions between IL-4 or IL-13 and the IL-4R*α* [80]. Although, FOXP3 is a characteristic marker of nTregs, Th3 cells can also be induced to generate FOXP3 [81–83]. Upon activation of the T cell receptor, Tregs suppress dendritic cells, B cells, macrophages, osteoblasts, mast cells, NK cells, NKT cells, CD4^+^ T, and CD8^+^ T cells [84]. 

Tregs suppressive mechanisms transpire through cytokine secretion, cytolysis, metabolic destruction, and altering of APCs function. The secretion of inhibitory cytokines such as IL-10, TGF-*β*, and IL-35 suppresses immune function. The inhibitory effects of IL-10 occur via its association with APCs to suppress inflammation hence, in the absence IL-10-secreting Tregs there is an increase in inflammation [85]. IL-35 on the other hand suppresses the expansion of T cells into other T helper subsets, as well as B cells and macrophages [86, 87]. TGF-*β* is necessary for the survival of Treg subsets [88]. Tregs may suppress the function of other cells via granzyme-mediated killing following the release of granzymes into the target cells [89, 90]. Similarly, metabolic disruption involving induction of adenosine and the production of cyclic adenosine monophosphate (cAMP) may be a vital mechanism for suppressing overreactive cells [91]. The versatility in Treg effector function allows them to modulate innate immune cells in particular APCs. This entails the engagement of surface molecules such as CTLA-4 and LAG-3 with MHCII molecules on the APCs conferring inhibitory responses that avert the stimulation of other conventional T cells [92]. 

As HSPs regulate an extensive component of the immune system, it is likely that they have a role in the optimal function of most immune cells. Importantly, the chaperoning effects of HSPs are necessary for the induction of certain T-cell phenotypes, importantly, Th1, Th2, and Tregs. This presupposes that HSPs are important in Treg function. To date, the following HSPs have been investigated in relation to Tregs, HSP60, HSP70, and HSP90. HSPs are important in the induction, proliferation, suppressive function, and cytokine production of Tregs. 

HSP60 employs TLR2-signaling pathway in regulating Treg function. TLR2 is expressed on the surfaces of Tregs [93] hence, association between the TLR2 on the Tregs and the HSP stimulates a sequence of events that affect the functional properties of Tregs. Incidentally, increasing levels of HSP60 are correlated with proportional elevations in the intensity of CD4^+^ CD25^+^ Treg-directed suppression on the production of TNF-*α* and IFN-*γ* [94]. An increase in HSP60 increases ligand binding of the HSP and the TLR2, thus, increasing suppression. This may represent an autoreactive inflammatory response causing autoimmunity [95]. HSP60 also causes an increase in Treg secretion of TGF-*β* and IL-10 [94]. HSP60 enhances the differentiation of cord blood cells into CD4^+^ CD25^+^ Foxp3^+^ Tregs [96]. Similarly, costimulatory signals from p277 also increase the activity of CD4^+^ CD25^+^ Tregs [94]. Therapeutic administration of HSP60 increases the presence of nTregs, and this is often correlated with a decrease in atherosclerotic plaques, the generation of Tregs, and an increase in the production of TGF-*β* [97, 98]. The concentration of HSP60 affects Treg suppression and proliferation. Hence, with respect to TLR2 on Tregs, strong ligand binding results in Treg proliferation while relatively low levels or interactions of ligands and TLR2 on the Treg result in an increase in Treg suppression [99].

Equally, HSP70 in Tregs promotes heightened suppressive function in Tregs [100]. HSP70 confers its activity via TLR4 pathway inducing a surge in the regulatory activities of Tregs. The TLR4-signaling pathway is important in Treg function, and this may be important for FOXP3 induction and suppression of inflammatory reactions [101]. TLR4 interactions with HSP70 may also augment effector T cell suppression by Tregs as this has been confirmed in coculture experiments with other ligands [102]. Additionally, the type of Tregs present following HSP administration may be dependent on the type of inflammatory response occurring at the time. For example in the mice model of arthrosclerosis, immunization with HSP70 produces a significant amount of CD4^+^ CD25^+^ Foxp3^+^ Tregs [97]. Similarly, adoptive transfer of HSP70 peptide epitopes such as B29 induces antigen-specific FoxP3^+^ or LAG-3^+^ CD4^+^ CD25^+^ Tregs that are effective in either aborting or suppressing arthritis in mice [103]. B29 is highly immunogenic peptides with conserved sequences that are presented to T cells by MHC II molecules. Immunization with HSP70 increases IL-10 producing Tregs [104]. HSP70 derived from mycobacterium tuberculosis stimulates the proliferation of Tregs by acting through dendritic cells causing a surge in IL-10 while dampening TNF-*α* release [105]. Additionally, HSP70 has anti-inflammatory properties including down-regulating inflammatory cytokine production, increasing cell and tissue tolerance of cytokine-related cytotoxicity, and influencing the permeability of the epithelial barrier [106]. 

HSP90 is important for conserving proteins involved in signal transduction, via a multichaperone complex [107]. HSP90 can be regulated by histone deacetylases (HDACs) such as HDAC6, and hypoacetylation of HSP90 occurs in the presence of excessive HDAC6 [108]. HDAC6 belongs to the Class II family of HDACs that are necessary for the removal of acetyl from histones and are found in the nucleus and cytoplasm [109]. In Tregs, the removal of HDAC6 results in the overexpression of HSP90 acetylation resulting in an increase in HSF1-related genes instigating an increase in the suppressive function of Tregs [100]. This may be important in treating patients with colitis. Mice deficient in HDADC6 are more likely to have increased levels of Treg suppression due to the presence of HSP90 and excess Foxp3 [100]. Similarly, mice deficient in HDAC9 have an increased expression of Foxp3 [110]. The presence of HDAC9 has been observed to decrease Foxp3 via deacetylation and incidentally Treg function. HSP70 not only acts via the TLR4 to regulate Tregs, but also may inhibit HDAC9 ultimately enhancing the release of Tregs and effective Treg repression [111]. Acetylation is a necessary posttranslational modification process for protein production. Hence, increased acetylation of Foxp3 may avert ubiquitination, increase its regulatory effects, stability, and promote DNA binding [112, 113]. Therapeutic strategies involving the use of HSPs to enhance the availability of Foxp3^+^ Tregs may be important in autoimmune diseases while in diseases like cancer it may be necessary to inhibit Foxp3 acetylation [112].

## 4. Conclusion 

In summary, despite the limited amount of research on Tregs and HSPs, the available literature suggests an involvement of HSPs in the suppressive function and cytokine production of Tregs. HSPs may indirectly or directly stimulate Tregs, via acetylation, TLR, ligation or act as costimulatory molecules via the induction of other cells or molecules to stimulate the Tregs. These may involve cytokines, antigens, and APCs. Hence, HSPs acting are therefore essential in inducing Foxp3 expression, cytokine secretion, and mediating Treg suppressive effects. Additionally, peptides such as B29 may have therapeutic potential as they are able to suppress inflammation and maintain tolerance. Thus, the therapeutic advantage of HSPs relates to their potential use in diseases where the function of Tregs is impaired, importantly, in the management of autoimmune diseases. 
